# Support Measures to Estimate the Reliability of Evolutionary Events Predicted by Reconciliation Methods

**DOI:** 10.1371/journal.pone.0073667

**Published:** 2013-10-04

**Authors:** Thi-Hau Nguyen, Vincent Ranwez, Vincent Berry, Celine Scornavacca

**Affiliations:** 1 Laboratoire d'Informatique, de Robotique et de Microélectronique de Montpellier, University Montpellier 2 - Centre national de la recherche scientifique, Montpellier, France; 2 Montpellier SupAgro (Unité Mixte de Recherche AGAP), Montpellier, France; 3 Institut des Sciences de l'Evolution de Montpellier, Unité Mixte de Recherche 5554, University Montpellier 2, Montpellier, France; 4 Institut de Biologie Computationnelle, Montpellier, France; University of Lausanne, Switzerland

## Abstract

The genome content of extant species is derived from that of ancestral genomes, distorted by evolutionary events such as gene duplications, transfers and losses. Reconciliation methods aim at recovering such events and at localizing them in the species history, by comparing gene family trees to species trees. These methods play an important role in studying genome evolution as well as in inferring orthology relationships. A major issue with reconciliation methods is that the reliability of predicted evolutionary events may be questioned for various reasons: Firstly, there may be multiple equally optimal reconciliations for a given species tree–gene tree pair. Secondly, reconciliation methods can be misled by inaccurate gene or species trees. Thirdly, predicted events may fluctuate with method parameters such as the cost or rate of elementary events. For all of these reasons, confidence values for predicted evolutionary events are sorely needed. It was recently suggested that the frequency of each event in the set of all optimal reconciliations could be used as a support measure. We put this proposition to the test here and also consider a variant where the support measure is obtained by additionally accounting for suboptimal reconciliations. Experiments on simulated data show the relevance of event supports computed by both methods, while resorting to suboptimal sampling was shown to be more effective. Unfortunately, we also show that, unlike the majority-rule consensus tree for phylogenies, there is no guarantee that a single reconciliation can contain all events having above 50% support. In this paper, we detail how to rely on the *reconciliation graph* to efficiently identify the *median* reconciliation. Such median reconciliation can be found in polynomial time within the potentially exponential set of most parsimonious reconciliations.

## Introduction

Gene families evolve through a complex process involving, among other things, incomplete lineage sorting and evolutionary events such as speciation (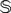), gene duplication (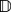), horizontal gene transfer (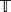) and gene loss (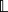). The resulting differences between the histories of gene families (gene trees) and the history of the species in which the genes are located (species tree) provide clues that are used by reconciliation methods to infer the events undergone by gene families. Accurately inferring those evolutionary events is essential in studying genome evolution as well as in inferring orthology relationships.

Reconciliation methods construct a mapping between a gene tree and a species tree to explain their incongruence by macroevolutionary events such as 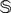,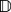,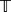, and 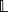. Several reconciliation methods have been recently developed according to the parsimonious or probabilistic paradigm (see [1] for a review). Parsimony methods search for a discrete evolutionary scenario of minimum overall cost according to the elementary cost assigned to each basic evolutionary event [2]–[8]. Probabilistic methods search for a continuous scenario maximizing the likelihood, or the posterior probability, of gene trees [9]–[11]. The latter methods are more realistic than parsimony methods, but their usage is limited to small sets of genes and taxa due to their high computing time. In contrast, parsimony methods can easily deal with tens of thousands of gene families [12].

A major issue with reconciliation methods is that the reliability of inferred evolutionary events may be questioned for several reasons: Firstly, there may be multiple equally optimal reconciliations for a given species tree - gene tree pair. Secondly, reconciliation methods can be misled by inaccurate gene/species trees [13]–[16]. Thirdly, predicted events may fluctuate with method parameters such as the cost of elementary events. This can lead to overestimating the number of evolutionary events, to erroneously annotate genes as being orthologous and overall to undermine the value and usage of reconciliation methods. All of these reasons highlight the need for methods to infer support values for evolutionary events predicted by reconciliation methods.

Recently, Park et al. [17] proposed a bootstrap based method for estimating the support of horizontal gene transfers in the phylogenetic network framework, regardless of duplications and losses. Considering the reconciliation problem involving duplications, transfers and losses (the DTL model), Scornavacca et al. [18] suggested to use a *reconciliation graph* (DTL-graph) to infer supports for evolutionary events based on their frequencies in the set of equally parsimonious reconciliations. However, no experiments have been carried out so far to assess the relevance of such supports. In this paper, we test this approach and complement it with a number of steps that increase the accuracy of inferred evolutionary events. For instance, when several most parsimonious reconciliations exist, we propose to return a median reconciliation rather than a random one, as done by state of the art methods. We define two variants of median reconciliations and provide polynomial algorithms for computing them. Experimental results show that such median reconciliations lead to significantly more accurate inferences in several situations. Median reconciliations are all the more appealing since there are cases where no parsimonious reconciliation can contain all events with high support (>50%). Hence, a pairwise compatibility of events does not ensure a global compatibility of those events.

Considering the whole set of equally parsimonious reconciliations is a first step toward the estimation of event reliability. Yet this is often not sufficient to provide accurate supports for evolutionary events. For instance, when there is a unique optimal reconciliation, the solution proposed in [18] is unadapted since, as the considered reconciliation set contains a single reconciliation, all its events have maximal support. Moreover, via simulations, it has been observed that the real evolutionary histories of gene families can slightly differ from the optimal reconciliations [14]. In such cases, suboptimal solutions may more accurately reflect the real evolution. This prompted us to study a method for inferring event supports from a set of (sub)optimal reconciliations obtained by computing most parsimonious reconciliations for slightly different elementary event (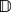,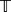,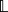) costs. Confidence values for evolutionary events are then computed according to their frequen cy among this set of sampled (sub)optimal reconciliations. As Doyon et al. [1] showed that most likely reconciliations are in the closed neighborhood of the most parsimonious ones, our strategy to obtain event supports can thus be seen as a rough approximation of event posterior probabilities. This approach is presented here in the parsimonious framework proposed by Doyon et al. [5] but it could easily be extended to the probabilistic framework.

Experiments on simulated data show the meaningfulness of the proposed support measures. Indeed, the evolutionary histories composed of events with high supports (*e.g.*≥50%) are more accurate than those proposed by traditional reconciliation tools, which do not use supports. Although such improvements were achieved for all the different support measures that we tested, measures accounting for suboptimal reconciliations perform significantly better than those that focus only on equally parsimonious reconciliations.

## Basics

This section outlines the prerequisites needed to fully understand how our method can, in polynomial time, assess event reliability and select a reconciliation containing the most supported events. After introducing the basic notations used in the reconciliation framework, we recall the formal definition of the parsimonious reconciliation problem with respect to the DTL model introduced by Doyon et al. [5] and present the graphDTL structure [18] that allows us to design a polynomial time complexity solution.

### Basic notations

**Figure 1:**
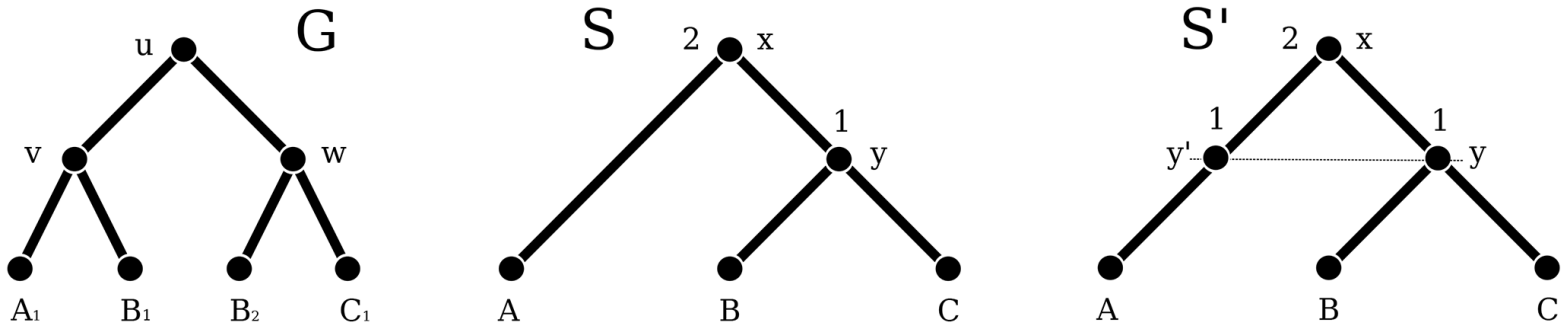
An example of trees. An example of a gene tree (*G*) and of a dated species tree (*S*), along with its subdivision (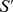). The species labeling of *G* is as follows: 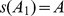, 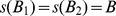 and 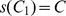.

The trees considered in this paper are binary rooted trees, labeled only at their leaves, and uniquely leaf-labeled (this simplifies definitions, while not keeping several leaves of a gene tree from corresponding to sequences of the same organism, see Figure 1). The node set, edge set, leaf node set and root of a tree *T* are respectively denoted *V*(*T*), *E*(*T*), *L*(*T*) and *r*(*T*). The label of each leaf *u* is denoted 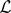
(*u*), while the set of labels of leaves of *T* is denoted 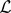
(*T*). Given two nodes *u* and *v* of *T*, we write 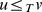 (resp. 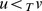) if and only if *v* is on the sole path from *u* to *r*(*T*) (resp. and *u*≠*v*). For a node *u* of *T*, 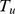 denotes the subtree of *T* rooted at *u*, 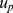 the parent node of *u*, hence 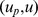 is the *parent edge* of *u*. When *u* has two children, they are denoted 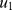 and 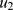. The *height* of *u*, denoted *h*(*u*), corresponds to the maximum number of edges along a direct path between *u* and any of the leaves in the subtree 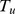.

A *species tree* is a rooted binary tree depicting the evolutionary relationships of ancestral species (internal nodes) leading to a set of extant species (leaves). A species tree *S* is considered here to be *dated*, that is associated with a time function 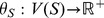 such that if 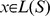 then 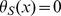 and if 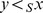 then 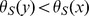. T he date of a node represents the time separating it from extant species. Such dates are usually expressed in million years and estimated on the basis of molecular sequences [19] and fossil records. To ensure that predicted transfers only occur between two co-existing species, absolute dates are not required, with the important information here being the relative order of the nodes of *S* induced by the dating. Given a dated binary species tree *S*, the reconciliation model we rely on considers a *subdivision* 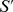 of *S* (as also done in [5], [10], [20]) together with an associated time function 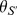. This subdivision is constructed as follows: for each node 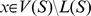 and each edge 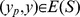 s.t. 
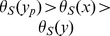
, a new node *w* is inserted along the edge 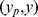, with 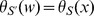. Moreover, for nodes 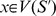 corresponding to nodes already present in *S*, we set 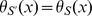.

A *gene tree G* is a rooted binary tree depicting the evolutionary history of a gene family, i.e. of a set of homologous sequences observed in current organisms. The sole label associated with each leaf of the gene tree, i.e.
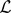
(), corresponds to a specific extant copy of the gene in a species. Note that several leaves of a gene tree can be associated with the same species due to duplication and transfer events. We define a surjective function 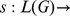 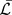 as the *species labeling* of *G*, where *s*(*u*) is used to denote the species to which the sequence *u* belongs. The set of species labels of the leaves of *G* is denoted 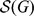. Each edge 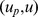 of *E*(*G*) can be univocally identified by the subset 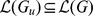. An example of a species tree and its subdivision, along with a gene tree is presented in Figure 1.

### Parsimonious reconciliations

**Figure 2:**
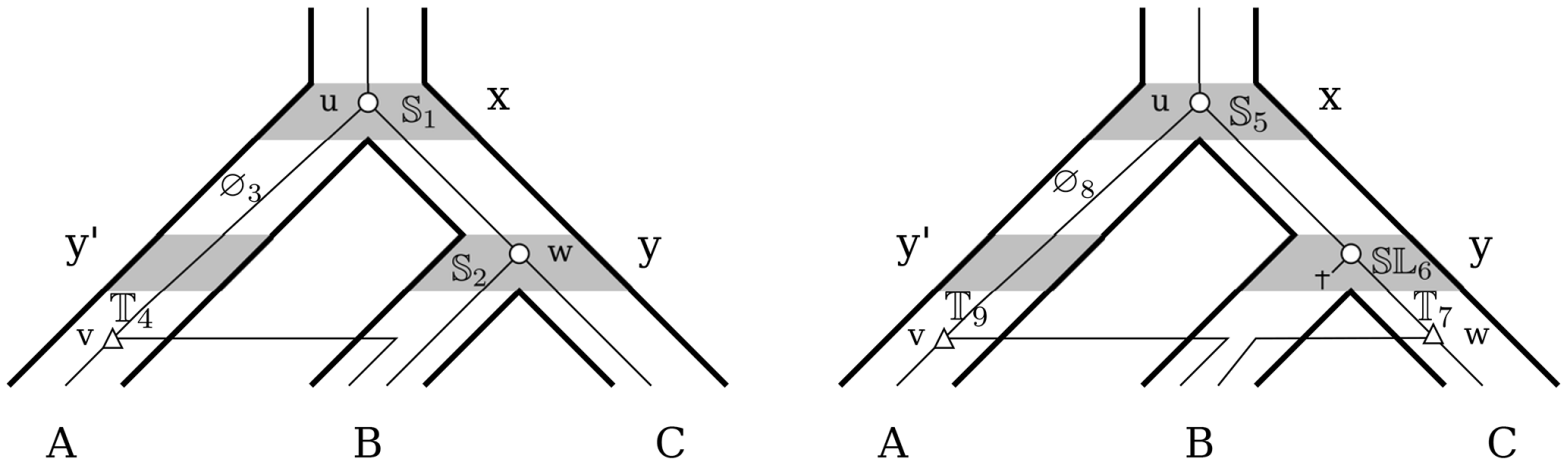
An example of reconciliation. Two valid reconciliations for the trees depicted in Figure 1 (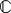 events are not indicated). The reconciliation on the left contains two 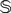 events, one 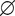 event, a 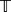 event and four 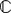 events, while the one 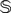 on the right contains one 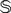 event, one 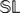, one 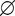 event, two 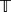 events and four 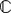 events.

Inspired by the work of several other authors, Doyon et al. [5] proposed a parsimonious reconciliation model for reconciling a dated binary species tree *S* with a binary gene tree *G* by building a mapping *α* that associates each gene 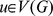 to an ordered list of nodes in *V*(*S*), namely the ancestral and/or extant species in which the sequence *u* evolved. This model takes four kinds of biological events into account: gene speciation, duplication, transfer and loss. To ensure the time consistency of transfers and to optimize the running time, the mapping is based on a set of seven atomic events: a speciation (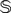), a duplication (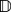), a transfer (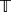), a transfer followed by loss of the non-transferred child (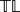), a speciation followed by loss of one of the two resulting children (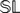), a no event (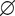) indicating that a gene lineage has crossed a time boundary, and a contemporary event (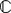) associating an extant gene copy with its corresponding species. For completeness, we reproduce the formal definition of a 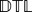 reconciliation [5] in Appendix S1. As an example, consider the reconciliation depicted on the left of Figure 2. This reconciliation corresponds to the following mapping *α*: 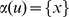 (event 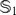), 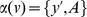 (events 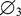 and 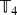), 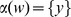 (event 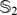), 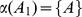, 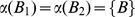 and 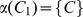. Note that several valid reconciliations can exist. For example, both reconciliations in Figure 2 are valid reconciliations for the trees depicted in Figure 1. Actually, given a gene tree *G* and species tree *S*, the number of possible reconciliations is infinite when successive 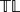 s are allowed, and still huge otherwise. Discrete evolutionary models compare alternative reconciliations by counting the number of events that these reconciliations respectively induce. As different types of event can have different expectancies (*e.g.* 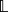 are thought to be more frequent than 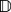 and 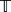 [21]), reconciliation models allow for a specific cost to be associated with each kind of event. The cost of a reconciliation *α* is then the sum of the costs of the individual events it induces, i.e. 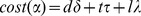, where *δ*, *τ*, and *λ* respectively denote the cost of a 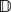,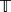, and 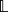 event, while *d*, *t*, and *l* respectively denote the number of these events in the reconciliation *α*. In this setting, the parsimony approach consists in preferring a reconciliation of minimum cost, called a *Maximum Parsimony Reconciliation* (MPR). Note that several distinct alternative reconciliations can have the same optimal reconciliation cost. Note also that distinct reconciliations on 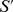 can be equivalent with respect to *S*, whereby one can identify a unique *canonical* reconciliation on 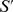 for each such equivalent reconciliation set [5], [18].

### The 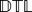 graph

In the reconciliation field, given a gene tree *G* and a species tree *S*, the main aim is to find the optimal reconciliation with respect to a chosen evolutionary model. One difficulty is that there can be several optimal or near-optimal reconciliations. In the maximum likelihood framework, numerous reconciliations may have a probability that is not significantly different from the optimal one, while in the parsimony framework there can sometimes be an exponential number of most parsimonious reconciliations [4]. Scornavacca et al. [18] introduced a compact bipartite graph to represent, in a common structure, a set of reconciliations on the basis of their shared events. This *reconciliation graph* (also called *DTL-graph*) is outlined below. This graph is an efficient solution to represent the set of MPRs as it has at most size 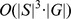 and can be constructed in 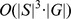 time from *G* and *S*, in spite of the possibly exponential size of the represented set. Moreover, a single traversal of the DTL-graph allows us to compute, for each event *e*, the number of MPRs displaying it and hence its frequency among *the* set of (canonical) MPRs reconciliations ([18], Section 4.2).

**Figure 3:**
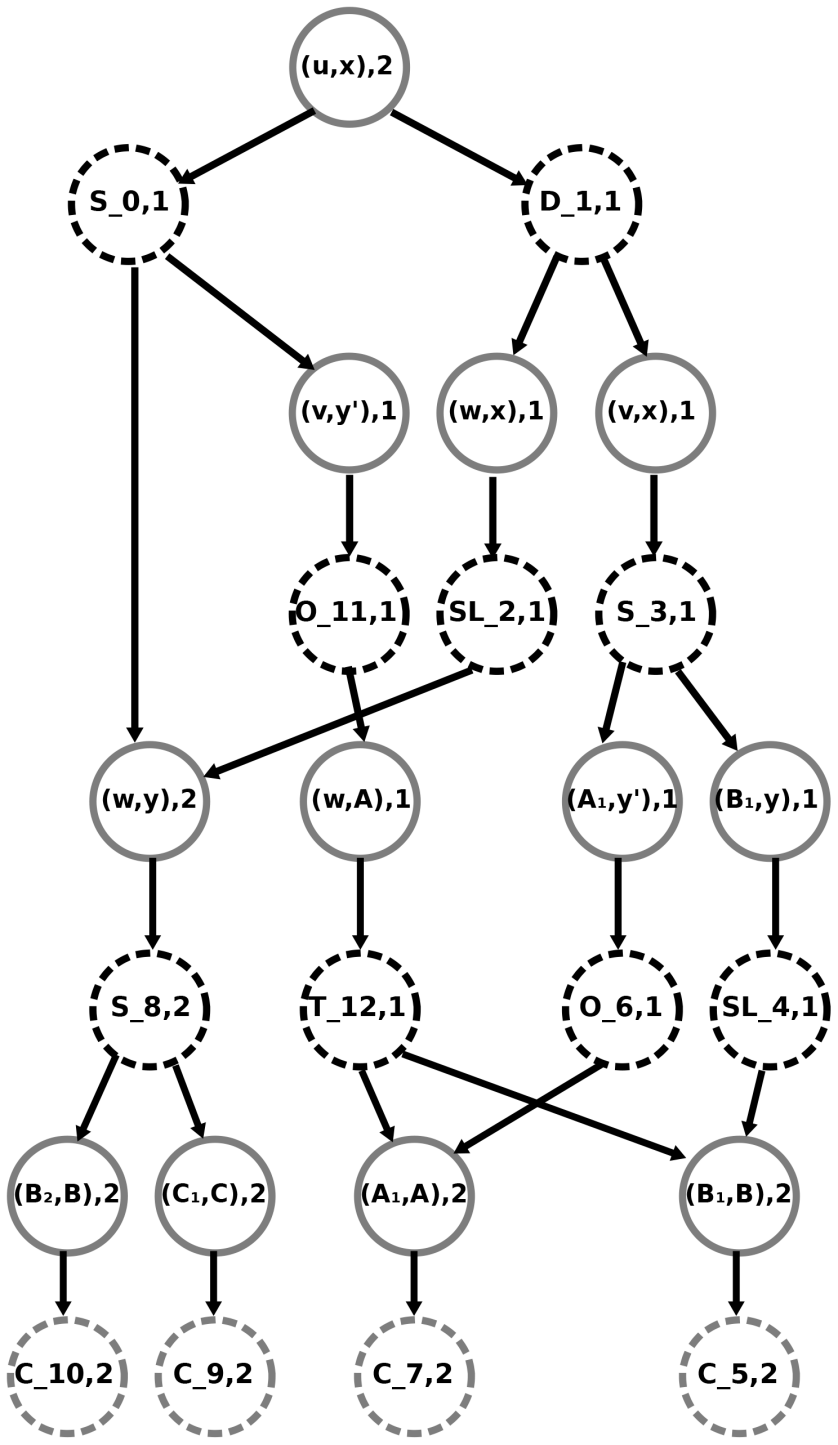
An example of a DTL graph. The DTL graph produced by Algorithm 3 of [18] for the trees depicted in Figure 1 and costs *δ* = 0.9, *τ* = 1.1 and l *λ* = 0.1. Event nodes are depicted using dashed lines and mapping nodes using solid lines.

More formally, a DTL-graph 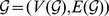 is composed of *mapping* nodes and *event* nodes, respectively denoted 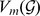 and 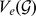. Each event node corresponds to an event (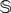,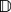,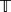, …), and each mapping node associates a node of *G* with a node of 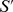. For instance, in Figure 3, the node denoted 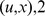 is a mapping node while the one denoted 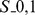 is an event node. In more detail, let 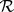 be the set of all MPRs for a gene tree *G*, the subdivision 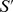 of a species tree, and a vector of costs of individual events. Then, for each 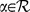, node 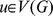 and index 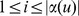 such that 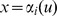, 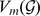 contains the node labeled 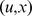. In particular, a *root* of 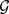 is a mapping node whose association concerns the root *r*(*G*) of *G* (note that 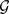 can have multiple roots). Moreover, two mapping nodes labeled 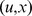 and 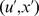 are connected via an event node labeled 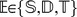 if and only if there exist 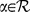 and index 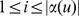 such that 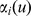 is associated with an event of type 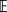 in Definition 1 in Appendix S1. and either (1) 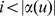, 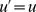 and 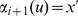 or 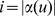, 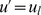 (or 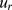) and 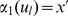 (or 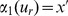). For instance, in Figure 3, the mapping node denoted 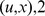 at the top of the graph associates the gene node *u* with the species node *x* while the nodes just below – denoted 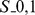 and 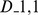 – indicates that *u* can be associated with *x* via a speciation (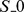) or a duplication (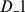). The values following the commas (2, 1 and 1, respectively) indicate the number of reconciliations encoded in the graph containing the nodes. Graph 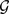 is constructed in such a way that each reconciliation 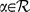 is depicted as a subgraph of 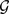 called the *reconciliation tree* 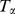 associated with *α*. By construction, 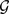 contains all MPRs of *G* and 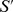. Moreover, all reconciliation trees in 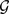 are associated with one reconciliation in 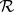, i.e. 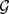 is a tight representation of 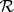. For further detail please refer to [18].

To introduce the notations needed hereafter, let *ch*(*u*) denote the set of children of a node *u* in 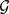. Moreover, for each event node *e* in 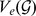, 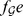 denotes its frequency in the set of *canonical* MPRs in 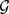, computed as described in [18], Section 4.2. We also call 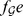 the MPR *support* of *e*, or simply *support* when the context is clear. Only canonical reconciliations are considered here when computing the frequencies of events to give the same weight to each event defined w.r.t. *S* (since each one may correspond to several events w.r.t. 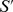 [5], [18]).

## Methods

In this section, we define the notion of median reconciliation, a reconciliation of choice to represent a set of reconciliations. We then detail how to efficiently compute this median reconciliation for the set of most parsimonious reconciliations by relying on the *reconciliation* graph introduced in the previous section. Finally we introduce a method to sample suboptimal reconciliations by altering the costs of elementary events and detail how the median reconciliation of this larger set of reconciliations can also be computed in polynomial time.

### Median reconciliations

When faced by the fact that several reconciliations can be optimal for the parsimony criterion, several methods and computer programs return a randomly chosen optimal solution, e.g. [7], [12], whereas CoRe-PA [22], Mowgli [5], the new version of Jane [20], and NOTUNG [8] only offer, as an alternative solution, to output all most parsimonious scenarios. Dealing with this list is not straightforward since there can be an exponential number of most parsimonious reconciliations [23]. When looking for a good representative of a set of objects, an intuitive choice is to select the median. Here we investigate the notion of the *median* of a set of reconciliations, proposing two variants of such a median. To define median reconciliations, we first need to specify distance measures between such objects.

Let *R*_1_ and *R*_2_ be two reconciliations 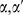 on the same gene tree *G* and species tree *S* whose respective event sets are denoted 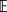 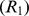 and 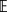 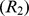. Now, let 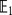 be an event in 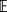 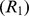 corresponding to the mapping 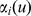 and let 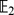 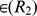 correspond to the mapping 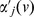. Then we have the following:

**Definition 1.** *We say that* 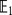 *is equivalent to* 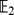 *, denoted* 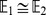 *, if and only if:*

1. *u* = *v*;

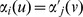
;
3. *one of the following conditions holds:*

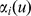
 (and thus 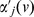) is a leaf;

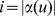
, 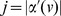, 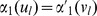 and 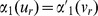 (or the symmetric holds);

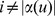
, 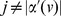 and 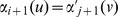.

The asymmetric distance between *R*_1_ and *R*_2_ is defined as:
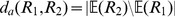
(1)while the symmetric distance 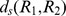 is defined as:
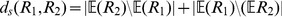
(2)


The first distance only accounts for events of *R*_2_ missing in *R*_1_, while the second distance also accounts for events in *R*_1_ not in *R*_2_. Note that, by definition, all reconciliations of a given gene and species tree pair have the same set of 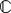
events, so these events will not be considered hereafter. As an example, let *R*_1_ and *R*_2_ be the two reconciliations depicted in Figure 2. For these reconciliations it holds that 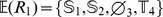 while 
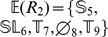
. Since 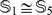, 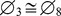 and 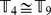, then 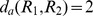 and 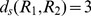. The above defined distances are the direct analogues of distances used in the phylogenetic reconstruction field: For instance, the symmetric distance is defined there as the number of clades (or splits in the unrooted context) present in one tree but not in the other [24]. In this field, there is a direct link between median trees and the support of clades (or splits) in a tree: when defining the support of a clade as the frequency of its appearance in a set of trees, the median tree happens to be the tree containing all clades with over 50% support, known as the majority-rule tree [24], [25]. If a more informative output is needed, one can rely on the asymmetric median tree, which is defined as the tree maximizing the sum of the frequencies of its clades, hence potentially including clades with lower than 50% support (see [26] for more details on consensus and median trees).

**Figure 4:**
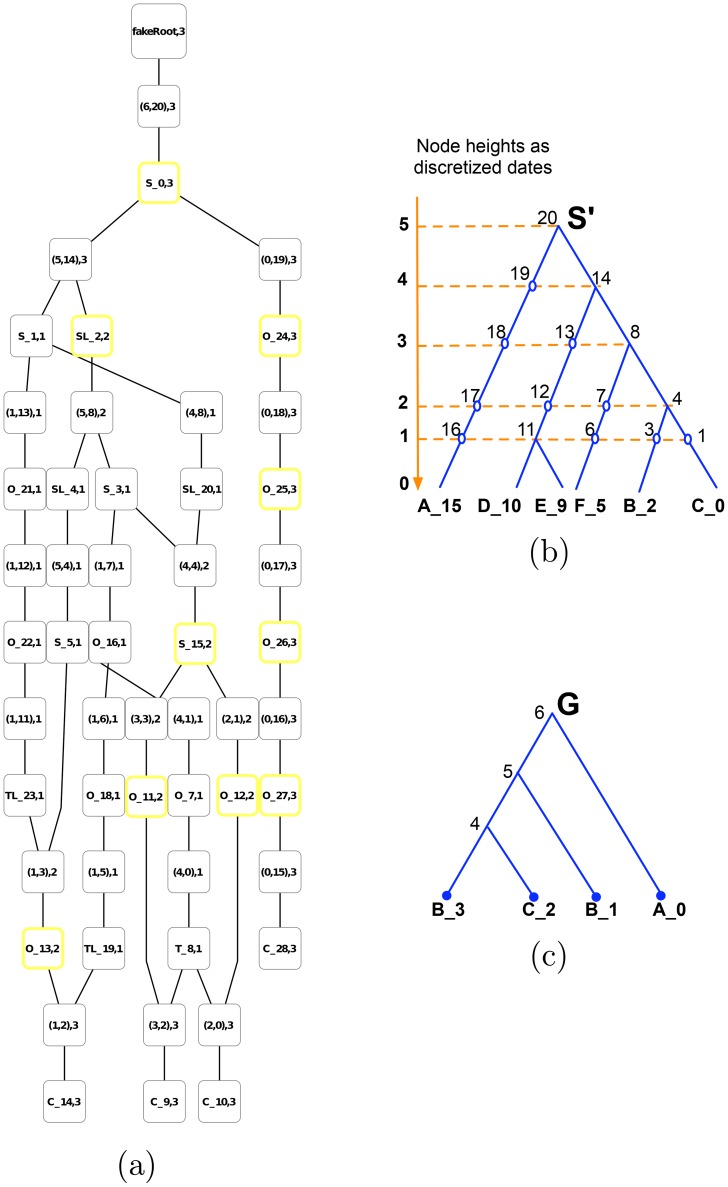
An example where none of the MPRs contain all highly supported events. (a) The DTL-graph composed of three canonical MPRs was computed by Algorithm 3 of [18] given the species tree *S* whose subdivision is 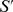 (b), the gene tree *G* (c), and the costs *δ* = 0.60205, *τ* = 0.74818, and *λ* = 0.24303 respectively for a 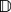,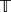, and 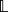
event. Events with support higher than 50% are highlighted by yellow squares. Each node of 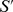 (resp. *G*) is assigned a unique id. An event node (resp. mapping node) of the graph is labeled as “
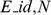
” (resp. “
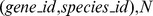
”), where 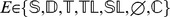, and *N* is the number of parsimonious reconciliations passing through the node. Recall that each parsimonious reconciliation tree can contain only one child of a mapping node.

Reconciliations are more complex objects than trees and unfortunately the set of events present in more than 50% of an input set of reconciliations cannot always be embedded in a single reconciliation. Indeed, Figure 4 shows a case where none of the most parsimonious reconciliation contains all events with above 50% support. In other words, the global compatibility of the set of events having above 50% support is not ensured. However, rather than resigning oneself to picking a random reconciliation, it seems preferable to select one with as many highly supported events as possible. This is why we turn to medians. Indeed, we will show in the next section that the medians of the reconciliation set used to estimate event supports are precisely the reconciliations with as many highly supported events as possible (see Lemma 1). To ensure that the proposed reconciliation is parsimonious, we limit our search to the input set, thus considering the problem of finding the “most median” reconciliation among input reconciliations, both in the asymmetric and symmetric case:

**Problem 1.** *Asymmetric Median Reconciliation (AMR)*

Input: *A set* 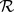 *of reconciliations on the same gene tree G and species tree S, such that* 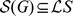 *.*

Output: *A reconciliation* 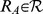 *minimizing* 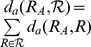 *over all reconciliations R in* 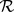.

**Problem 2.** *Symmetric Median Reconciliation (SMR)*

Input: *A set* 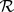 *of reconciliations on the same gene tree G and species tree S, such that* 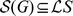 *.*

Output *: A reconciliation* 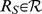 *minimizing* 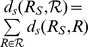 *over all reconciliations R in* 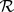.

Note that there can be several reconciliations within the initial set 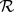 minimizing 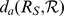 or 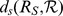. In the worst case, all reconciliations of 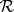 can have the same value for those functions, thus returning one of the (a)symmetric medians of 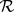 is just equivalent to returning a random reconciliation of 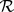. Such problematic cases occur, for instance, when reconciliations have no events in common. In these extreme cases, it does not really matter which reconciliation is chosen since all of its events will have a low support (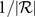). Moreover, in most realistic cases, only one or a few reconciliations will minimize 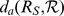 (or 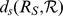), and the (a)s-median criterion will allow us to select, among MPRs, the one with the most frequent (i.e. reliable) events.

### Computing median reconciliations

We now explain the link between the frequencies of events in a set of reconciliations and the criteria optimized by a median reconciliation of this set. Given a reconciliation set 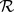, 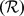 denotes the set of events that appear in at least one reconciliation in 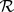. Given an event 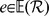, *f*(*e*) denotes the frequency of this event in 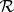, i.e. the proportion of reconciliations displaying *e*.

**Lemma 1.**

- *The asymmetric median reconciliation* 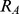 *of a set* 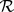 *of reconciliations is one of the reconciliations maximizing* 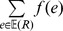 *, over all reconciliations R in* 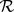 *.*
- *The symmetric median reconciliation* 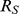 *of a set* 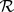 *of reconciliations is one of the reconciliations maximizing* 
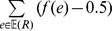
 *, over all reconciliations R in* 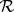 *.*

**Proof:** Deferred to Appendix S1.

Note that though the two criteria stated in Lemma 1 seem quite similar, they generally do not lead to choosing the same reconciliation as representative of 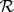. As an example, let *R*_1_ and *R*_2_ be the two reconciliations depicted in Figure 2. These reconciliations *R*_1_ and *R*_2_ have equal event sets except for 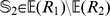 and 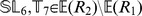. Suppose that 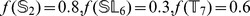. Then 
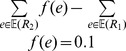
 leading *R*_2_ to be preferred for the asymmetric median, while 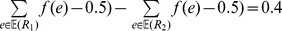 leading *R*_1_ to be preferred for the symmetric median.

### Polynomial time algorithms to identify median reconciliations of most parsimonious reconciliations

Note that, since there can be an exponential number of MPRs, median reconciliations cannot be constructed in polynomial time from a raw representation of the set of all MPRs (indeed, this would require exponential running time just to read this input set). We get around this problem thanks to the DTL-graph representation of the set of MPRs – that can be computed and stored in polynomial time and space. We now show how to compute asymmetric and symmetric median reconciliations for a set of reconciliations depicted by a DTL-graph in polynomial time. Let 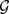 be the DTL-graph for a gene tree *G* and the subdivision 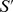 of a species tree containing all MPRs 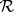 of *G* and 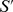. Recall that solving the AMR problem is to find the reconciliation 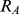 minimizing 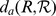 = 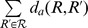 over all reconciliations 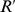 in 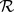, so, by Lemma 1, the one maximizing 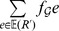, over all reconciliations 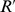 in 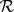. Here, each reconciliation 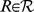 corresponds to a reconciliation tree 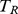 in 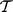, and obtaining *R* from 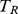 is straightforward ([18], Algorithm2). We will then focus on identifying a reconciliation tree for which the sum of event supports is maximized. This is achieved by a single traversal of 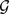, described in the Algorithm below (see Table 1 and Table 2). Note that from a practical standpoint of view, it suffices to subtract 0.5 to all event supports in a preprocessing step to transform Problem 2 into Problem 1. So the Algorithm can also be used to solve Problem 2, having previously subtracted 0.5 from all event supports.

**Table 1:** Algorithm 1: maxSumFrequenciesTree 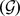.

1 **for** 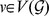 **do**
2 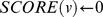 ; // score of the best local reconciliation encountered so far for 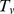
3 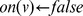 ; // whether or not this node is part of the global optimal reconciliation tree
4 **for** 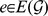 **do**
5 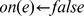 ; // whether or not this node is part of the global optimal reconciliation tree
6 **for** *each vertex v of V* (  G) *in post-order* **do**
7 **if** *v* is an event node **then**
8 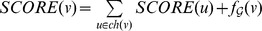 ;
9 **else**
10 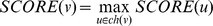 ;
11 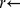 a root of 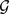 such that 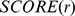 is maximum among all roots of 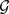 ;
12 backtrack(*r*);
13 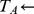 the subtree of 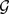 obtained by keeping nodes and edges which are “on”;
14 return 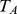 ;

**Table 2:** Algorithm 2: backtrack(*v*).

1 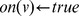 ;
2 **if** *v is an event node* **then**
3 **for** *any outgoing edge e of v* **do** 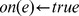 ;
4 **for** *each child u of v* **do**
5 backtrack(*u*);
6 **else**
7 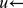 a child of *v* such that 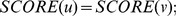
8 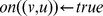 ;
9 backtrack(*u*);

We first prove the correctness of the algorithm.

**Theorem 1.** *Let* 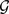 *be the minimum reconciliation graph for a dated species tree S, a gene tree G such that* 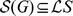 *and positive costs δ, τ, and λ for a* 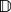 *,* 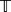 *, and* 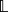 *, event respectively. Algorithm 1 (* *Table 1* *) extracts a reconciliation tree* 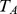 *from* 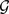 *such that the sum of the event supports of* 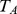 *is maximum among all reconciliation trees included in* 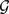 *, i.e. among all MPRs.*

The proof is deferred to Appendix S1.

**Theorem 2.** *Algorithm runs in* 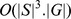 *time.*

**Proof:** Since each node *v* of 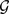 is considered on line 8 or 10 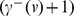 times, where 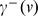 is the number of edges entering in *v*, the overall complexity of lines 1–10 is proportional to 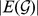. The subroutine backtrack(*r*) constructs a reconciliation tree by a pre-order traversal of the subgraph of 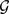 rooted at *r*. Since each node of this subgraph is considered at most once by construction, the overall complexity of this step and of Algorithm 1 (Table 1) is 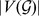. Both 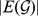 and 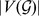 are bounded by 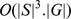 (Theorem 2 of [18]). This concludes the proof.

Overall, the above results show that we can easily compute central representatives of most parsimonious reconciliations between a gene and species tree.

### Considering suboptimal reconciliations by altering the elementary event costs

The choice of the cost for the elementary events may have a strong impact on the event set inferred by parsimonious reconciliation methods. These costs are usually derived from evolutionary event rates inferred by probabilistic methods on biological datasets [11]. In the case of simulated datasets, exact event rates are known and can be directly used to derive elementary event costs (see [5], [27] and Equation 4).

A standard strategy to estimate the reliability of an inference is to consider its stability with respect to fluctuations of the method parameters, i.e. here the costs of the elementary events (see [28] for an example in the sequence alignment context). Since optimal solutions for slight variations of parameter values are near-optimal solutions for the original parameter values, this strategy can also be viewed as a sampling of suboptimal solutions. To obtain a set of Near-optimal Parsimonious Reconciliations (NPRs), we thus proceeded as follows: first, we fixed a value for a parameter, denoted Δ, controlling the dispersion of new elementary costs. Second, for each elementary event type *E* (with *E* being 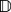,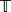 *,or* 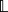,), a new cost 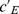 was randomly drawn from a Gaussian distribution with mean equal to the initial cost 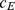, and standard deviation equal to 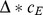, i.e. 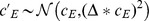. Third, the resulting combination of elementary costs was input in Algorithm 3 of Scornavacca et al. [18] to construct a DTL-graph 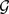 that summarizes the MPRs for this parameter set. These MPRs, for a set of altered costs, can be seen as NPRs for the original parameter set. The last two steps were repeated 1,000 times without varying the value of Δ, producing a set 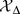 of 1,000 DTL-graphs summarizing the set of generated NPRs.

The support of an event among NPRs can then be defined as the percentage of NPRs containing it. In practice, the NPR-based support of an event *e* can be computed by combining its MPR supports observed in the 1,000 DTL-graphs as follows:
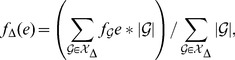
(3)where 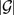 denotes the number of MPRs encoded in 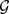. Having computed such global NPR supports and assigning them to the corresponding node event in each DTL-graph in 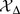, the symmetric and asymmetric median reconciliation problems can be solved by running Algorithm 1 (Table 1) successively on the 1,000 DTL-graphs of 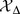 and selecting the best overall returned reconciliation. Hence, considering NPRs instead of MPRs just increases the running time by a constant factor, without increasing the asymptotic time and space complexity. Note that the definition of NPR-based supports indeed generalizes the MPR-based one, since when Δ tends to 0, NPRs tend to MPRs. Indeed, if Δ = 0, then 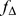 is just the aforementioned 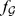 support.

### Filtering out unreliable events

Both support measures considered above, i.e. computing the frequency of events either from MPRs or from NPRs, can be used to filter a reconciliation event set by retaining only events whose frequency is not smaller than a given threshold (called *filtering threshold* in the following). This may be used to prune poorly supported events from proposed reconciliations. A very similar process is applied in phylogenetics where branches whose support is lower than a chosen confidence threshold are discarded, being considered as unreliable. In phylogenetics, a clade is removed by collapsing the edge of the tree above the clade, with the filtering process still outputting a (partially resolved) tree. With reconciliations, this is not so simple, as there is no guarantee that the events having a threshold above 50% together form a reconciliation. Thus, such event subsets have to be considered as a partial history of events, still allowing us to interpret part of the gene tree (with some of its nodes being assigned to these events). This however suffices to deduce orthology and paralogy relationships among some leaves or to qualify some edges of the gene tree as representing a transfer.

## Results and Discussion

In this section, we report an experimental evaluation of the ideas outlined in the previous sections to answer several questions. Mainly, how can an optimal reconciliation be selected when several are available? Does filtering out the least supported events in a reconciliation improve the accuracy of the inference? Does considering near optimal reconciliations as well as optimal ones lead to more reliable support estimates?

### Generating data

Experiments were conducted on the basis of a phylogeny of 37 proteobacteria. Along this species tree, 1000 evolutionary histories, composed of 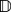, 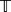 *,* 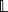 *and* 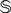 and events (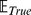), were simulated according to a birth and death process, leading to 1000 simulated gene trees (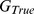). Rates for macro-evolutionary events were chosen using the same scheme as [14]: (a) the loss rate was randomly chosen in the [0.001, 0.0018] interval, where the units are events per gene per million years. Moreover, the ratio between the “birth” rate (sum of the duplication and transfer rates) and the loss rate was randomly chosen in the [0.5,1.1] interval, while the proportion of the duplication rate to the birth rate was randomly chosen in the [0.7,1] interval. The Seq-Gen program [29] has been used to simulate the evolution of DNA sequences of 1500–3000 bp length along each 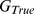 under the Generalised time-reversible (GTR) model [30], the sequences in turn have been given as input to RAxML [31] to infer a maximum likelihood gene tree (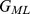). Thus, the simulation protocol delivered a dataset of 1000 gene trees to reconcile with the proteobacteria phylogeny. ML trees contain on average 29 leaves and have an average Robinson-Foulds distance of 17.7% with respect to the true gene trees. The species tree is reconciled with 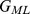 trees instead of 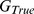 trees to take the fact that gene trees are only an indirect estimation of the true gene histories into account. For more details on the simulation protocol please refer to [14].

As done in [14], the initial elementary cost for a duplication was chosen as follows:
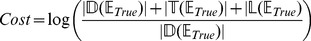
(4)where 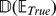, respectively 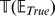 and 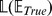, stands for the sets of duplication, respectively transfer and loss events, in the simulated history 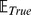. The elementary costs of a transfer and of a loss, 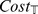 and 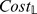, were computed in the same way. Speciation events were not penalized, *i.e.* 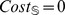, as often done [5], [7], [12].

### Compared strategies to infer events in gene histories

We tested the relevance of several event prediction *strategies*, on the basis of four choices:

- Which set of reconciliations to choose from: the set containing the most parsimonious reconciliations only, or a broader set containing non-optimal ones computed by altering the value of event costs given as input to the reconciliation algorithm via the Δ parameter, see previous section. In the experiments, we studied Δ values in the 0%–40% range, i.e. going from strictly optimal to loosely optimal parsimonious reconciliations.
- How to compute the support 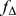 for events of the selected reconciliation set, i.e. on the basis of MPRs only (Δ = 0) or also from NPRs (Δ = 10% to 40%);
- How to pick a reconciliation among those of the selected reconciliation set, i.e. a random one, the asymmetric or the symmetric median reconciliation;
- Under which *T* threshold to filter out events from the chosen reconciliation. In the experiments, we considered the following filtering thresholds: *T* = 100%, *T* = 90%, 50% and *T* = 0%. Note that the last case corresponds to applying no filter at all.

To test the above mentioned strategies, for each gene family (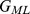), we used Algorithm 3 of Scornavacca et al. [18] to compute a reconciliation graph 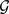 containing all MPRs. We first did this using the event costs computed by Equation (4) – that we consider to be our best candidate for the “real” costs – and then did this for altered values of these costs (according to the noise level Δ), giving rise to a graph containing more and more non-optimal reconciliations (NPRs) as increasing Δ values were used (see Section *Considering suboptimal reconciliations by altering the elementary event costs*).

A note on the running time. For each gene family, computing the 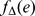 support for all events took at most 15 min, while computing the median reconciliations took only a few seconds.

### Measuring the accuracy of compared strategies

In order to compare the performance of those event prediction strategies, we studied the accuracy of the resulting predicted events with respect to those of the true (simulated) history. Following Def. 1 a 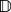 event of 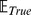 is said to be correctly predicted (i.e. is a true positive or TP) when the reconciliation places the corresponding node of 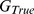 on the correct branch of the species tree. Similarly, a 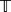 event is said to be correctly predicted when the corresponding edge of 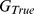 goes from the same donor to the same receiver branch of the species tree as in the correct gene history. An 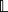 event is correctly predicted by a reconciliation when it is placed in the species tree branch where it occurred in the true history of the family. A predicted event absent from 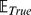 is a false positive event (*FP*), while events which are not within the inferred set of events are either true negatives (*TN*) if they are not in 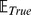 or false negatives (*FN*) otherwise. For 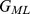, only the type of an event (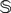, 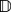, 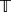, or 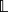) and its predicted location on the species tree are taken into account in the computation of TP, FP, TN and FN values (i.e. the location in the gene tree is disregarded).

As done in previous papers [5], [14], the reconciliation error was measured on 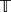, 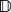 and 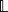 events, i.e. events causing a gene tree to differ from the species tree. The error of a predicted set 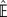 estimating an event set 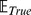 is then measured by the symmetric evolutionary distance between these sets:
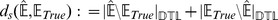
where the first and second term respectively correspond to *FP* and *FN*, and where the 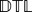 subscript recalls the events taken into account. This simple measure was used on the dataset composed of 1000 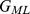 families to compare the competing strategies to estimate a gene true history, a strategy being all the more accurate when its average error is low.

To obtain a more detailed comparison between competing strategies, one often resorts to a Receiver Operating Characteristic curve (ROC-curve), allowing us to represent the performances of alternative methods on a number of datasets in a single 2D graphic. As we currently do not have any practical solution to compute the number of true negatives (*TN*) for the problem considered here, we considered Precision-Recall curves (PR-curves) instead. PR-curves are very similar to ROC-curves [32] and can be drawn while disregarding *TN*. Precision and Recall values are defined as follows:
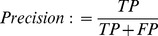
(5)

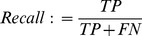
(6)Each competing strategy gives rise to a PR curve, which are then compared on a single common plot. According to (5) and (6), the higher the PR curve is for a given recall level, the better accuracy the corresponding method displays.

## Results

### Filtering out the least supported events increases the accuracy

We first focus on strategies for filtering events of a randomly chosen MPR. Table 3 reports the average error performed by such strategies depending on whether all the events in the random MPR are considered, or only those appearing in at least *T* percent of the MPRs, i.e. events for which 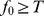. We tested several filtering thresholds *T*, namely 0%, 50%, 90% and 100%. Note that *T* = 0% corresponds to the behavior of current reconciliation tools, which do not use supports.

**Table 3:** Impact of event filtering on random MPRs.

Filtering Threshold *T*	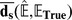	FP	FN	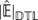
0	11.3	8.9	2.4	20.2
50%	**10.9**	8.2	2.7	19.2
90%	**10.4**	7.5	2.9	18.2
100%	10.4	7.5	2.9	18.2

This table shows the accuracy of filtering events from a random MPR, when varying the filtering threshold *T*. The event supports have been computed from MPRs only, *i.e.* Δ = 0. Thus, for each line, the set 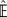 contains all events *e* from the chosen random MPR having 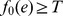. Column 2 (resp. Column 3 and 4) reports the accuracy as measured by the average symmetric distance 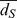 (resp. FP and FN) between 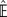 and 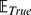. A bold-faced value indicates that the accuracy of the corresponding strategy is significantly better than that of the previous row (p-values of the paired *t.tests* are lower than 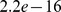). Column 5 reports the average numbers of predicted, 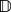, 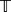, and 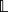 events with or without filtering. On average, the true evolutionary history of a gene family contains 13.7 such events.

Table 3 shows that the support values computed through the tool presented in [18] allow us to filter out one to two events on average (where the unfiltered 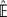 contains on average 20.2 events). An analysis of the *FP* and *FN* components of the error shows that three out of four times the removed events are indeed erroneous events. The total error thus decreases from 11.3, when no filtering step is applied (*T* = 0%), to 10.4, when keeping only the events present in all MPRs (*T* = 100%). Note that each filtering statistically leads to a significant reduction in the distance between the predicted and true event sets, as compared to a less restrictive filtering (bold-faced numbers in the table). The only exception is when going from *T* = 90% to *T* = 100%, since both strategies output the same event set 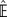.

Although being lowered by the filtering process, one can wonder why the error is at such a non-negligible level. Looking at the large number of events in an unfiltered reconciliation (right column of first row in Table 3) provides an explanation: with 20.2 events in 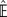 on average compared to 13.7 events in 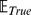, the predicted reconciliation contains many more events than the correct one. Yet as parsimony is penalized for each extra event it proposes, it tries to propose as few events as possible. Thus, it is much more likely that the gap between the sizes of 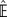 and 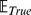 is due to errors in the gene trees. Indeed, each wrong branch contained in 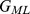 leads to contortions in the reconciliation to explain the discrepancy with the shape of the species tree. This matches findings of a previous paper showing that the inference error of reconciliations grows exponentially with the distance between the estimated and correct gene trees [14]. Looking in detail at *FP* shows that among the 20.2 events present in a full reconciliation, only half are correct, which indicates that a good filtering process is indeed needed before exploiting the inference results, e.g. to decide the orthology or paralogy of current sequences. The pattern observed for *FN* shows that even though 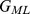 trees may be an imprecise estimation of the true gene trees, MPRs usually exhibit most of the correct events. Indeed, a random MPR misses only 2.4 of the 13.7 correct events. The good news is that the filtering process only loses a small proportion of these correct events, with *FN* increasing from 2.4 to 2.9 (going from *T* = 0 to *T* = 100%).

### Low variability in events among MPRs

The Table 3 results confirm the relevance of the filtering process proposed by Scornavacca et al. [18]. However, this filtering strategy filters out very few events. The l ast column of the table shows that on the ≈20 events composing a random reconciliation, ≈18 on average have a support of 100%. This implies that on average there is very low variability in the event sets from one MPR to another. F urther analysis reveals that for 53% of the 1000 gene families there is only a single MPR. In such cases, all event have a 100% support value, thus preventing any filtering. Note that even in cases where several MPRs are available, the variability among their respective event sets is relatively small: different MPRs on average share at least two thirds of their event sets. Indeed, relying on half of the dataset to explain an average gap of 2 events (20.2-18.2) between a full reconciliation and one containing only events of maximum support, shows that even in cases where several MPRs are available, they share at least 11 events over the 18 they contain on average. The significant reduction in the distance between filtered events and the true reconciliation observed in Table 3 is then obtained thanks to only half of the considered gene families. This shows that this approach is very powerful but also indicates that there is room for further improvement.

The low average variability of events among different MPRs leads to many events having maximum support; together with the fact that for *T* = 100% filtered event sets still contain too many events (18.2 compared to 13.7), this explains that filtering at the extreme *T* = 100% value leads to the lowest error rate. Moreover, the low variability among MPRs also explains why no significant difference in accuracy was observed in choosing an MPR at random or one of the two median reconciliations described earlier on in the paper (data not shown, p-value = 0.7223 and 0.1689): these strategies usually propose the same reconciliation, and otherwise propose very close event sets. This is a call to examine more elaborate ways to determine the support of events, and to consider larger reconciliation sets. For both of these tasks, more variability needs to be introduced among considered reconciliations. For this, we will resort to NPRs.

### Considering near-optimal reconciliations indeed increases the variability

Recall that nearly optimal reconciliations (NPRs) can be obtained by reconstructing most parsimonious reconciliations along with noisy event costs. Increasing the noise level Δ allows us to more broadly sample the suboptimal reconciliation space, and incidentally to consider new events: the set of MPRs spans 57.3 events on average, while 76.7 (Δ = 10%), 119 (Δ = 20%) and 430 (Δ = 40%) events are spanned when considering 1000 NPR graphs. Inferring the support of an event on the basis of its frequency among near-optimal reconciliations in addition to optimal ones generates more variability in the obtained event supports. As Table 4 shows, even for a moderate noise level (Δ = 10%), there is a significant difference in the average size of 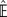 when varying the filtering threshold: 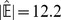 (*T* = 100%, Table 4) compared to 17.8 (*T* = 50%, Table 4) and to 20.2 when no filtering is applied (*T* = 0%, Table 3). These differences are to be compared with the small filtering effect that was observed in Table 3. More significant differences are obtained for higher noise levels (Δ = 20 and Δ = 40%). Fixing the filtering threshold allows us to measure the variability introduced when increasing the Δ noise level: fewer optimal reconciliations are obtained and the support of events is thus progressively reduced, as shown by smaller events sets being proposed. For example, for *T* = 50%, 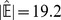 if Δ = 0% (Table 3) and drops to 17.8 when Δ = 10% (Table 4) and drops further to 5.6 when Δ = 40% (Table 4).

**Table 4:** The accuracy of event prediction strategies when inferring the support of events in MPRs from sets of NPRs.

Filtering threshold *T*	Noise level (Δ)	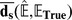	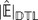
50%	10%	10.0	17.8
	20%	9.1	16.2
	40%	10.6	5.6
90%	10%	8.5	15.2
	20%	7.0	11.5
	40%	11.8	2.6
100%	10%	7.1	12.2
	20%	7.4	7.4
	40%	12.6	1.1

This table shows the average symmetric distance (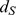) between predicted and true event sets when computing event supports 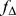 from their frequency in optimal and near-optimal reconciliations, and filtering the events with 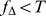 (Column 3), where *T* is the filtering threshold. The degree of non-optimality in reconciliations is indirectly measured by the noise level Δ introduced in the event elementary costs. Column 4 reports the average number of predicted 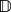, 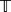, and 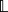 events, depending on both the filtering threshold and noise level. On average, the true evolutionary history of a gene family contains 13.7 such events.

### Accounting for near-optimal reconciliations provides more reliable event supports

Considering NPRs increases the chance of finding correct events that are absent from MPRs, but at the risk of both introducing incorrect events and lowering the support of correct events already present among MPRs. However, as Table 4 shows, the overall effect on the error terms is positive. The new supports are more reliable in the sense that filtering out events with low supports when the supports have been computed from NPRs decreases the inference error more significantly than when computing supports from MPRs only: the minimum error is 10.4 in Table 3, but decreases to 7.1 (Δ = 10%, *T* = 100%) and 7.0 (Δ = 20%, *T* = 90%) in Table 4. Compared to the 11.3 error level of the state of the art reconciliation methods (first row of Table 3), an overall improvement of 38% is achieved when combining the idea of filtering events and computing the event support by sampling near-optimal reconciliations. Thus, the latter idea really allows us to have a more accurate estimation of the inferred event robustness.

Note that there is a limit in the level of noise that is useful to introduce in the event costs, i.e. in the level of non-optimality to consider: the exaggerated Δ = 40% noise level always leads to higher error terms than those obtained when computing supports on the basis of MPRs alone (compare row 3, respectively 6 and 9 of Table 4 with row 2, respectively 3 and 4 of Table 3).

Thus, we can conclude that for a reasonable noise level, considering NPRs is a successful idea: having more reliable support values – and possibly also considering new correct events – easily offsets the fact that more erroneous events may be considered (most of these additional erroneous events are probably detected and filtered out thanks to the filtering step).

### Filtering non-optimal reconciliations leads to more accurate event sets

Relying on MPRs only to infer a set of events seems too restrictive, as shown by the results in Table 5. This table provides the accuracy results when selecting a symmetric, asymmetric, or a random reconciliation not only among MPRs but also considering NPRs. Comparing results of Tables 4 and 5 indeed shows that selecting a reconciliation at random from NPRs instead of MPRs alone almost systematically decreases the error, e.g. from 9.1 (row 2, Table 4) to 8.9 (row 4, Table 5) or from 8.5 (row 4, Table 4) to 8.4 (row 5, Table 5). The same trend is generally observed when selecting the symmetric and asymmetric median reconciliations (data not shown).

**Table 5:** The accuracy of strategies for selecting events from NPRs depending on various parameters.

		as-median NPRs		random NPRs		s-median NPRs	
Filtering Threshold *T*	Δ	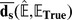	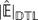	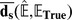	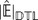	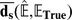	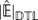
0%	10%	11.3	20.3	11.3	20.1	**10.1**	17.7
	20%	11.8	20.0	11.5	20.3	11.5	16.0
50%	10%	10.0	18.0	9.8	17.4	**8.2**	14.9
	20%	9.3	15.9	8.9	15.7	**7.5**	10.9
90%	10%	8.5	15.2	8.4	15.1	**7.7**	13.5
	20%	7.1	11.4	7.0	11.4	7.1	9.4

Parameter *T* denotes the filtering threshold and Δ denotes the noise level for generating sets of NPRs. Columns 3, 5, and 7 report the accuracy as measured by the average symmetric distance 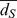 to the event set in the true gene history. Note that the bold-faced 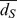 values indicate the method (among (a)s-median and random NPRs) having the symmetric distances of 1000 gene families 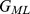 being significantly less than that of the other methods, i.e. p-value of the paired *t.test* being less than 0.05. Columns 4, 6 and 8 report the average numbers of predicted 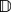, 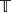, and 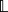 events with or without filtering. On average, the true evolutionary history of a gene family contains 13.7 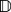,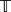, and 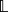 events.

### Other remarks on the filtering thresholds when considering NPRs

Rows 1 and 2 in Table 5 show that when no filtering is applied (i.e. *T* = 0%), broadening the event set by considering fewer optimal reconciliations (i.e. increasing Δ) only leads to fewer accurate reconciliations, whatever reconciliation is kept – random, symmetric or asymmetric median. This is confirmed for Δ values greater than 20% (data not shown). This results from the fact that more and more erroneous events are considered when the deviation from the original cost (measured by Δ) increases. Overall, considering NPRs introduces variability in reconciliations, which is very useful for filtering out incorrect events as we have discussed above, but applying no filtering amounts to retaining only the cons of the increased variability.

Another remark concerns the extreme filtering threshold (*T* = 100%). Results for this threshold were not included in Table 5 as they are identical among all reconciliation selection methods and identical to those displayed in Table 4. The latter point indicates that once supports are established from NPRs, selecting a reconciliation among MPRs or among NPRs is the same thing if we focus on events with 100% support. Indeed, each MPR event with 100% support must be found in at least one NPR among the 1000 replicates performed to sample near-optimal reconciliations. Thus, in our experiments, all MPR events are most likely also NPR events. Moreover, for the same reason, no new event with 100% support can appear in an NPR and not be referenced in MPRs. Lastly, note that such an extreme threshold leads to results of a quite good accuracy – 7.1 to 7.4 for Δ = 10% to 20%, Table 4 – compared to that of the 7.0 and 7.1 of the last row in Table 5.

### Advantages of symmetric median reconciliations

Table 5 also allows us to compare event inferring strategies on the basis of the procedure they use to select a reconciliation among NPRs.

The asymmetric median procedure selects on average a reconciliation with the same accuracy as the random selection procedures for *T* = 0%, 90% and 100%, but is significantly worse for *T* = 50%. By maximizing the sum of the events in the chosen reconciliation, the asymmetric median rather chooses a larger set of events – although each can be individually of lower support than events in another reconciliation (see example detailed in the Methods section). This is illustrated by the fact that the size of the predicted event set (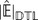 columns, Table 5) is almost always larger for asymmetric medians than when choosing a reconciliation by another procedure. This behavior might penalize the asymmetric median, as all the results reported above show that only events with a quite high support can be trusted. For *T* = 90%, the asymmetric median performs similarly to choosing a reconciliation at random as the events it specifically proposes are usually more poorly supported, hence have been filtered out. For *T* = 100%, all methods constantly output the same event set, so no difference can be observed in their accuracy.

The symmetric median procedure is the only reconciliation selection procedure that displays a significantly better accuracy than other procedures. As the bold-faced values in Table 5 are significantly lower than other terms in the row (at the 95% confidence level), it can be seen that in four out of the six studied conditions, the symmetric median procedure outperforms the other two. The only case where its accuracy is lower than another procedure is for *T* = 90% and Δ = 20%, where its error reaches 7.1 compared to 7.0 displayed by the random selection procedure – but this difference is not statistically significant.

### More detailed accuracy profile of competing strategies

**Figure 5:**
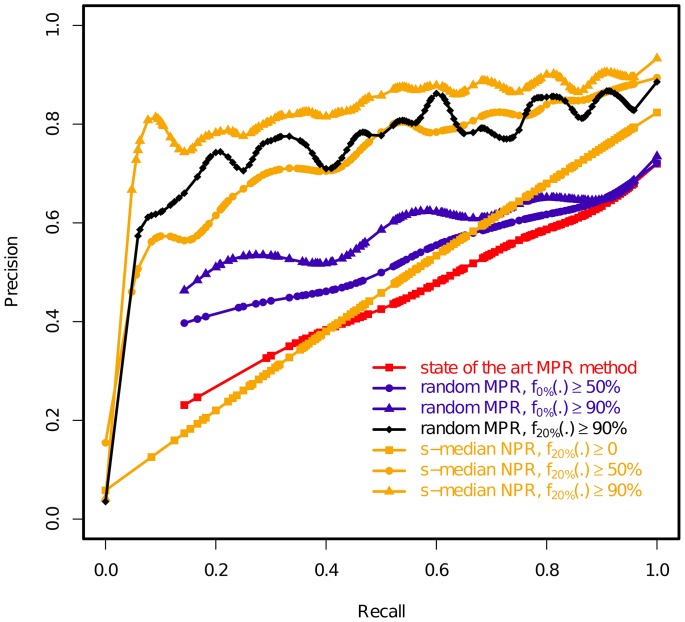
The accuracy of competing strategies to infer events in a gene history. Curves are plotted from experiments on 990 gene families. Strategies are defined by a considered reconciliation set – most parsimonious reconciliations (MPRs) or near-optimal reconciliations (NPRs), by a way to select one of these reconciliations – at random or through the s-median procedure, by the method to compute event supports 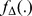
–Δ = 0% (i.e. computing supports from MPRs) or 20% (i.e. computing supports from NPRs obtained for a noise level of 20% in event costs), and by the subset of events output depending on their support (≥0%, ≥50% or ≥90%). See main text for a description of the proposed strategies.

Finally, Figure 5 represents the PR curves corresponding to the main event prediction strategies we mentioned above: 1) outputting a random MPR and applying no filtering (approach of the *state of the art methods*); 2) filtering events of a random MPR when computing supports from MPRs only (as proposed in [18]) with 50% and 90% filtering thresholds (curves *random MPR* 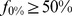 and *random MPR* 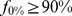); 3) filtering events of a random MPR when computing supports from NPRs (curve *random MPR* 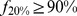); 4) selecting an *s-median* reconciliation among NPRs obtained for Δ = 20%: one curve for the strategy outputting all events in such a reconciliation (curve *s-median NPR* 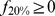), then two curves for strategies combining all the ideas presented in this paper (i.e. selecting an s-median reconciliation among NPRs, computing support from NPRs and filtering low-support events), for 50% and 90% thresholds (curves *s-median NPR* 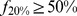 and ≥90%). Displayed curves are interpolations obtained on the inferences done on 990 gene families – ten out of the 1000 gene families contained no 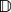, 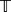, and 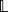 events having support above the studied thresholds, hence were excluded to avoid undefined values in the Precision and Recall computation.

This visual representation of event prediction accuracy highlights several important points. First, when outputting event sets corresponding to a full reconciliation – but leading to a similar average accuracy (see previous sections) – the profiles of the strategy choosing a random reconciliation (curve 1) is different from that choosing an s-median one (curve 5), i.e. the two methods will likely propose different event sets in general.

Filtering out the least supported events usually has a beneficial effect, as can be observed by comparing curve 1 to curves 2 and 3. On the basis of all the experiments, it can be concluded that the lowest symmetric evolutionary distance is obtained when fixing a high filtering threshold (e.g. 90%).

The most striking feature of Figure 5 is the supremacy of strategies filtering low support events while relying on NPRs to compute the support (curves 4, 6 and 7) which for the same recall level reach a much higher precision level than strategies not filtering events and a significantly higher precision level than strategies relying on MPRs only to compute event supports. In other words, the proportion of correct events among inferred events is substantially increased by such a combination of techniques. Note that the best results are obtained when Δ = 20% and *T* = 90%, but other combinations not studied here could give even better results.

### Remarks on the dataset of the true gene trees

Although considering only randomly chosen reconciliations, Doyon et al. [5] showed that parsimony reconciliation methods can correctly recover large parts of the true evolutionary histories of gene families when the true gene trees are given as input. This is confirmed on our dataset where the existence of multiple optimal solutions is also taken into account. In fact, among ≈44 events predicted per gene family when using 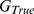 instead of 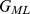, 94% of them are present in all MPRs, i.e. having 100% support, and 96% of these latter events are correct. Hence, inferring event supports from a set of nearly-optimal reconciliations will likely introduce wrong events. In our opinion, our proposed approach should be applied only when the performance of reconciliation methods is degraded due to erroneously constructed gene or species trees.

## Conclusion

In this paper, we achieved several goals:

Firstly, we showed the importance of not focusing only on *one* random optimal reconciliation. Indeed, given a gene and species tree to be reconciled, we introduced the median reconciliation concept to best summarize a set of reconciliations by choosing a central reconciliation rather than a random one. We provided algorithms to compute median reconciliations in polynomial time. In the experiments, the symmetric median reconciliation often performed significantly better than the strategy of choosing a random most parsimonious reconciliation. We showed the benefit of considering *all* optimal reconciliations to compute a simple support measure for each event in an inferred reconciliation. The tool provided by Scornavacca et al. [18] here nicely plays its role in managing, in polynomial time and space, the potentially exponential number of such reconciliations. Moreover, we showed that filtering out the least supported events significantly reduces the inference error. Finally, we showed how near-optimal reconciliations can be obtained and how sampling such reconciliations allows us to compute more reliable supports than those obtained by just considering most parsimonious reconciliations.

When aiming to estimate a set of events shaping a gene family history, the combined ideas discussed in this paper achieved an overall 38% increase in accuracy, as compared to the practice of considering just a single optimal reconciliation. This leaves little doubt on the use of the support values presented here. Lastly, we would like to stress that when focusing on a particular gene tree node, e.g. to decide on the orthology or paralogy of extant sequences, the support seems to be a reasonable estimate of the node's robustness. Our first experiments indicate that only events showing the highest support should be trusted, and that 90% and 100% filtering thresholds should be considered. Further study is needed to fully understand the link between this support measure and the confidence level in a statistical test, as for instance studied for bootstrap values in the phylogenetic context [33]–[35].

An implementation of the algorithms presented in this paper will be provided in the new version of the graphDTL software, available at http://mbb.univ-montp2.fr/MBB/subsection/downloads.php?section=all.

## Supporting Information

Appendix S1
**The formal definition of a** 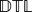 **reconciliation** [**5**] **and the proofs of Lemma 1 and Theorem 1.**
(PDF)
