# Supplementary material for: Support Measures to Estimate the Reliability of Evolutionary Events Predicted by Reconciliation Methods
Source: PLoS One. 2013 Oct 4;8(10):e73667. doi: 10.1371/journal.pone.0073667 (PMC3790797; doi:10.1371/journal.pone.0073667)
Supplement: Appendix S1 — The formal definition of a reconciliation [5] and the proofs of Lemma 1 and Theorem 1. (PDF) [file pone.0073667.s001.pdf]

Appendix S1

Formal definition of a reconciliation [5]

Definition 1. *Consider a gene tree G, a dated species tree S such that $\mathcal{S}(G) \subseteq \mathcal{L}(S)$, and its subdivision S'. Let α be a function that maps each node u of G onto an ordered sequence of nodes of S', denoted $\alpha(u) = (\alpha_1(u), \alpha_2(u), \ldots, \alpha_\ell(u))$. Function α is said to be a* reconciliation *between G and S' if and only if exactly one of the following events occurs for each pair of nodes u of G and $\alpha_i(u)$ of S' (denoting $\alpha_i(u)$ by x' below):*

a) if x' is the last node of $\alpha(u)$, one of the cases below is true:

1. $u \in L(G)$, $x' \in L(S')$ *and* $s(x') = s(u)$; ($\mathbb{C}$ *event*)
2. $\{\alpha_1(u_l), \alpha_1(u_r)\} = \{x'_l, x'_r\}$; ($\mathbb{S}$ *event*)
3. $\alpha_1(u_l) = x'$ *and* $\alpha_1(u_r) = x'$; ($\mathbb{D}$ *event*)
4. $\alpha_1(u_l) = x'$, *and* $\alpha_1(u_r)$ *is any node other than* x' *having height* $h(x')$ *or* $\alpha_1(u_r) = x'$, *and* $\alpha_1(u_l)$ *is any node other than* x' *having height* $h(x')$; ($\mathbb{T}$ *event*)

b) otherwise, one of the cases below is true:

5. x' *is an artificial node and* $\alpha_{i+1}(u)$ *is its only child;* ($\varnothing$ *event*)
6. x' *is not artificial and* $\alpha_{i+1}(u) \in \{x'_l, x'_r\}$; ($\mathbb{SL}$ *event*)
7. $\alpha_{i+1}(u)$ *is any node other than* x' *having height* $h(x')$. ($\mathbb{TL}$ *event*)

Proof of Lemma 1

Given a reconciliation R and an event e, let $ind(R, e)$ be the indicator function for e in R, i.e. $ind(R, e) = 1$ if $e \in \mathbb{E}(R)$ and $ind(R, e) = 0$ otherwise. Let R_A be the reconciliation of $\mathcal{R}$ minimizing

$$\begin{aligned} d_a(R_A, \mathcal{R}) &= \sum_{R \in \mathcal{R}} d_a(R_A, R) \\ &= \sum_{R \in \mathcal{R}} \sum_{e \in \mathbb{E}(R)} \big(1 - ind(R_A, e)\big) \\ &= \sum_{e \in \mathbb{E}(\mathcal{R})} f(e).|\mathcal{R}|.\big(1 - ind(R_A, e)\big) \\ &= \sum_{R \in \mathcal{R}} |R| \; - \; |\mathcal{R}| \sum_{e \in \mathbb{E}(R_A)} f(e) \end{aligned} \tag{1}$$

where $|\mathcal{R}|$ and $|R|$, respectively denote the number of reconciliations in $\mathcal{R}$ and the number of events in a reconciliation R. The claim for the asymmetric case then follows from the fact that the first sum and the $|\mathcal{R}|$ factor in (1) are independent of the choice of R_A.
Now for the symmetric distance, suppose R_S is a candidate reconciliation for being the symmetric median of $\mathcal{R}$, then for every event $e \in \mathbb{E}(\mathcal{R})$ each $R \in \mathcal{R}$ containing the event contributes by adding one to $d_S(R_S, \mathcal{R})$ if $e \notin \mathbb{E}(R_S)$, and each $R \in \mathcal{R}$ not containing the event contributes by adding one if $e \in \mathbb{E}(R_S)$. More

precisely, we have

$$\begin{aligned}
d_S(R_S,\mathcal{R}) &= |\mathcal{R}| \sum_{e\in\mathbb{E}(\mathcal{R})} \Big((1-f(e))ind(R_S,e) + f(e)(1-ind(R_S,e))\Big) \\
&= |\mathcal{R}| \sum_{e\in\mathbb{E}(\mathcal{R})} f(e) + |\mathcal{R}| \sum_{e\in\mathbb{E}(\mathcal{R})} \Big(ind(R_S,e).(1-2f(e))\Big) \\
&= |\mathcal{R}| \sum_{e\in\mathbb{E}(\mathcal{R})} f(e) + |\mathcal{R}| \sum_{e\in\mathbb{E}(R_S)} \Big(1-2f(e)\Big) \\
&= \sum_{R\in\mathcal{R}} |R| \;-\; 2|\mathcal{R}| \sum_{e\in\mathbb{E}(R_S)} \big(f(e)-0.5\big) \qquad (2)
\end{aligned}$$

This holds because R_S is in $\mathcal{R}$. The first summation term and the $2|\mathcal{R}|$ factor do not depend on the choice of R_S, hence the reconciliation minimizing $d_S(R_S,\mathcal{R})$ is that maximizing $\sum_{e\in\mathbb{E}(R_S)}\big(f(e)-0.5\big)$. $\square$

Proof of Theorem 1

Proof: For each node v of $\mathcal{G}$, we introduce the notion of *best local reconciliation support* for v, denoted $BLS\,(v)$, which corresponds to the maximum support achievable for event nodes of a subtree rooted at v and belonging to a reconciliation tree:

$$BLS\,(v) = \max_{\substack{T\in\mathcal{T},\\ v\in V_e(T)}} \left(\sum_{w\in V_e(T_v)} f_{\mathcal{G}}(w) \right) \qquad (3)$$

We will now show that $SCORE\,(v) = BLS\,(v)$, for each node $v \in V(\mathcal{G})$, which will prove the theorem as *i)* each root of $\mathcal{G}$ corresponds to the root of a reconciliation tree; *ii)* there is a bijection between $\mathbb{E}(R)$ and $V_e(T_R)$; i.e. line 11 will then be shown to return a suitable reconciliation tree.

The proof that $SCORE\,(v) = BLS\,(v)$ for each node $v \in V(\mathcal{G})$ proceeds by induction on the height of v. If $h(v) = 0$, by construction of $\mathcal{G}$, v is an event node such that $e(v) = \mathbb{C}$ [18] and, by line 8 of Algorithm 1, $SCORE\,(v) = f_{\mathcal{G}}(v) = BLS\,(v)$, as v has no child here. Let us now suppose that $SCORE\,(u) = BLS\,(u)$, for each node $u \in V(\mathcal{G})$ with $h(u) < h_i$ and let v be a node in $\mathcal{G}$ such that $h(v) = h_i$. Note that, if v is an event node, from Condition C_4 of Definition 5 of [18], each reconciliation tree in $\mathcal{T}$ containing v also contains all child nodes of v (that have a height strictly smaller than h_i). Thus:

$$BLS\,(v) = \max_{\substack{T\in\mathcal{T},\\ v\in V_e(T)}} \left(\sum_{w\in V_e(T_v)} f_{\mathcal{G}}(w) \right) = f_{\mathcal{G}}(v) + \sum_{u\in ch(v)} \max_{\substack{T\in\mathcal{T},\\ u\in V_e(T)}} \left(\sum_{w\in V_e(T_u)} f_{\mathcal{G}}(w) \right)$$

$$= f_{\mathcal{G}}(v) + \sum_{u\in ch(v)} BLS\,(u) = f_{\mathcal{G}}(v) + \sum_{u\in ch(v)} SCORE\,(u) = SCORE\,(v)$$

where these equalities hold by definition of $BLS\,(v)$, by induction and by line 8 of Algorithm 1. On the contrary, if v is a mapping node, from Condition C_5 of Definition 5 in [18], each reconciliation tree from $\mathcal{T}$ containing v also contains exactly one child node of v. Hence, $BLS\,(v) = \max_{u\in ch(v)} BLS\,(u) = \max_{u\in ch(v)} SCORE\,(u)$ $= SCORE\,(v)$, which holds by definition of $BLS\,(v)$, by induction and by line 10 of Algorithm 1. This concludes the proof that $SCORE\,(v) = BLS\,(v)$ for each node $v \in V(\mathcal{G})$ and thus ensures that node r selected on line 11 of Algorithm 1 maximizes $BLS\,(\cdot)$ among all roots of $\mathcal{G}$.

Algorithm 2 simply traverses $\mathcal{G}$ starting from the root node $r(T_A)$ of an optimal reconciliation tree T_A and identifies all other nodes of T_A. Indeed, the subset of nodes selected by Algorithm 2 satisfies all conditions

of Definition 5 of [18], and can thus be proved to be a valid reconciliation tree T_A using a proof similar to that of Theorem 1 of [18]. Moreover, it is straightforward to see that $BLS\left(r(T_A)\right) = \sum_{w \in V_e(T_A)} f_{\mathcal{G}}(w)$ and, since all reconciliation trees in $\mathcal{T}$ are rooted at roots of $\mathcal{G}$ [18], this concludes the proof. $\square$
